# Omega-3 Fatty Acids Effects on Inflammatory Biomarkers and Lipid Profiles among Diabetic and Cardiovascular Disease Patients: A Systematic Review and Meta-Analysis

**DOI:** 10.1038/s41598-019-54535-x

**Published:** 2019-12-11

**Authors:** Zuhair S. Natto, Wael Yaghmoor, Heba K. Alshaeri, Thomas E. Van Dyke

**Affiliations:** 1 Department of Dental Public Health, Faculty of Dentistry, King Abdulaziz University, Jeddah, Saudi Arabia; 2 Department of Periodontology, School of Dental Medicine, Tufts University, Boston, MA USA; 3 Department of Oral Health Policy and Epidemiology, School of Dental Medicine, Harvard University, Boston, MA USA; 4 Department of Oral Sciences, Faculty of Dentistry, Umm Al-Qura University, Jeddah, Saudi Arabia; 5 Department of Pharmaceutical Sciences, Fakeeh College of Medical Sciences, Jeddah, Saudi Arabia; 6 Center for Clinical and Translational Research, The Forsyth Institute, Cambridge, MA USA; 7 Department of Oral Medicine, Infection and Immunity, School of Dental Medicine, Harvard University, Boston, MA USA

**Keywords:** Cardiology, Diabetes

## Abstract

The purpose of this systematic review and meta-analysis was to investigate omega-3 fatty acids’ influence on 12 inflammatory biomarkers—LDL, HDL, total cholesterol, TG, HbA1c, Apo AI, Apo AII, Apo B, CRP, TNF-α, glucose, and fasting blood glucose among diabetic and cardiovascular disease (CVD) patients. We searched articles in six database engines, and 16 of the 696 articles reviewed met the inclusion criteria. Among these, lipid and inflammatory biomarkers investigated commonly included total cholesterol (11 studies), LDL, and TG (10 studies each). Overall, omega-3 was associated with a significant reduction in Apo AII among diabetic patients, as compared to different controls (−8.0 mg/dL 95% CI: −12.71, −3.29, *p* = 0.0009), triglycerides (−44.88 mg/dL 95% CI: −82.6, −7.16, *p* < 0.0001), HDL (−2.27 mg/dL 95% CI: −3.72, −0.83, *p* = 0.002), and increased fasting blood glucose (16.14 mg/dL 95% CI: 6.25, 26.04, *p* = 0.001). Omega-3 also was associated with increased LDL among CVD patients (2.10 mg/dL 95% CI: 1.00, 3.20, *p* = 0.0002). We conclude that omega-3 fatty acids may be associated with lower inflammatory biomarkers among diabetic and cardiovascular patients. Clinicians should be aware of these potential benefits; however, it is essential to recommend that patients consult with clinicians before any omega-3 intake.

## Introduction

Chronic inflammation is the primary characteristic of several diseases, including diabetes and cardiovascular disease (CVD)^1,2^. Type 2 diabetes leads to hyperglycemia, which affects leukocyte counts, in addition to polymorphonuclear neutrophil (PMN) and monocyte function through several mechanisms. These include the production of advanced glycation end products (AGEs), increased extracellular superoxide dismutase release, and such proinflammatory cytokine secretions as interleukin-1 beta (IL-1), sialic acid, insulin-like growth factor (IGF), C-reactive protein (CRP), tumor necrosis factor alpha (TNF-α), and matrix metalloproteinase (MMP)^1,3^.

Elevated levels of several inflammation markers, such as C-reactive protein (CRP), fibrinogen, and various cytokines have been reported in CVD studies^4–7^, and when these markers’ levels are reduced, CVD’s severity decreases^8^.

In healthy individuals, both inflammation’s onset and resolution should be efficient, and turning off inflammation signals should be associated with the loss of pro-inflammatory factors. One way to accomplish this is to use specialized immunoresolvents molecules, such as resolvins, lipoxins, protectins, and maresins, that mediate the resolution of inflammation^1,9,10^. This approach helps the body return to homeostasis through active and highly regulated “programmed resolution”. These mediators trigger the pathways that signal the physiologic end of the acute inflammatory phase in several diseases^9,11^^,^^12–18^.

The topic of this systematic review is omega-3 fatty acids and pro-resolving lipid mediators’ effects on inflammatory biomarkers and lipid profiles. Pro-resolving molecules can be divided into 4 groups. The first includes lipoxin (LX) from endogenous metabolism, and arachidonic acid (AA), which promotes healing via receptor agonists and controls the resolution phase of acute inflammation^19–21^. The second includes resolvins and, more recently, protectins and maresins, which are derivatives of dietary omega-3 polyunsaturated fatty acids (PUFAs)^1,11,22–24^. These molecules share similar pro-resolving characteristics to LX and receptor agonists^20,25^, and provide a promising new approach to control inflammation by focusing on enhancing the “off signal” rather than simply inhibiting the “on signal”^25^. It also decreases the likelihood of side effects associated with the conventional anti-inflammatory treatments available^4,20^. Several studies have investigated these lipid mediators’ role in resolving inflammation which they found significant improvements in total antioxidant capacity (TAC), and nitric oxide (NO) with significant reduction in malondialdehyde (MDA). No changes were observed in levels of glutathione (GSH), superoxide dismutase (SOD) or catalase (CAT)^26,27^. However, we do not know yet omega-3 fatty acids’ precise effects on certain lipid profiles and inflammatory biomarkers among diabetic or CVD patients, which are the subject of this review. The PICOS were as follows: P (Population), diabetic or CVD patients; I (Interventions), any form of omega-3; C (Comparisons), any placebo control or a comparison group or diet; O (Outcomes), inflammatory biomarkers; and S (Study Design), randomized clinical trials.

## Methods

This study followed the Preferred Reporting Items for Systematic Reviews and Meta-Analyses (PRISMA) guidelines^28^ (S1 Text). The study protocol is available in the Supporting Information section (S2 Text). The protocol was registered prospectively in PROSPERO (CRD42015015961).

### Search strategy and inclusion criteria

To be included, randomized controlled studies had to provide information about omega-3 fatty acids, eicosapentaenoic acid (EPA), docosahexaenoic acid (DHA), or one of the following lipid mediators: lipoxins (lipoxin A4, B4), resolvins (resolvin E1, E2, D1), protectin (D1, AT-PD1), or maresin (maresin 1). These studies also had to be conducted with diabetic or CVD patients who received at least 1,000 mg of omega-3 fatty acids. Using a search strategy that combined terms (see S3 Text), two investigators (ZN, WY) screened MEDLINE, Web of Science, Embase, the Cochrane Central Register of Controlled Trials, and Scopus,. In addition, internet search engines (e.g., Google Scholar) were screened from 1980 to January 31, 2018. Any type of treatment that used the previous form of omega-3 fatty acids or mediators was eligible for inclusion in this review, as were all healthcare interventions and outcomes using the focused mediators. We enhanced the database searches using the following approaches: (1) screening bibliographies of full-text articles included; (2) searching the citations of selected articles on Google Scholar and Web of Science, and (3) contacting authors and experts in the field for additional articles.

### Exclusion criteria

We focused primarily on omega-3 polyunsaturated fatty acids’ effects on lipid profiles and inflammatory biomarkers. Therefore, we did not consider dose-response or noncompliance. We excluded studies that used any omega-3 fatty acids derived from an alpha-linolenic acid (ALA), which has different sources and mechanisms. Although our search was not restricted by language, we did not identify any articles that required translation or were in a language unknown to the authors.

### Data extraction and quality assessment

We extracted data independently in duplicate (ZN, WY). Any discrepancies were resolved through discussion or by a third reviewer when necessary. The following information was collected: country; study type; lipid mediators; area; total sample size; gender; age; duration; outcome; population; test or control type; dose, and mean and standard deviation (SD) before and after the intervention, or mean and SD differences.

The studies’ quality was assessed with respect to potential bias using the Cochrane Collaboration’s tool that assesses the risk of bias (Higgins 2011). Two independent authors (ZN and WY) assessed the quality and discrepancies, which were resolved through discussion. Our conclusions are summarized in the Risk of Bias section (S4 Text).

### Statistical analyses

A meta-analysis was conducted using mean differences (MDs) and their SDs for continuous outcomes, or these values were calculated using the data available. For studies that did not include SDs, a correlation coefficient of 0.5 was used based on Follmann *et al*.’s method. The results were combined across studies using a random-effects model. All units of measurement were converted into a single standard across the studies (mg/dL). The results were summarized with forest plots using the I-squared (I^2^) statistic for heterogeneity. The primary outcomes were changes in inflammatory biomarkers across all groups, including LDL, HDL, total cholesterol, TG, HbA1c, Apo AII, CRP, and TNF-α. The analyses were reported for each disease (diabetes and CVD).

Sensitivity analyses were attempted based on data availability, results, and the incorporation of aggregate data from different control studies and durations. Funnel plot asymmetry was applied to consider possible publication bias (S5 text).

All analyses were conducted using Review Manager (RevMan) v. 5.3 (Cochrane Collaboration, London, UK). The data were presented as means with SDs and 95% confidence intervals (CI).

## Results

**Figure 1:**
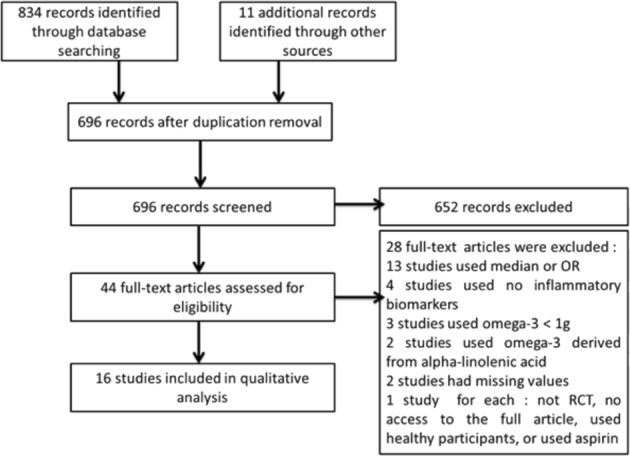
PRISMA Flow diagram of the numbers of studies screened and included in the review.

Of the 696 unique studies from the combined searches, 44 studies were assessed in full and 16 were deemed eligible (Fig. 1). We excluded certain studies for the following reasons: the authors used the median or odds ratio (OR) (13 studies); no inflammatory biomarkers were used (4 studies); less than 1000 mg/day of omega-3 fatty acids was administered (3 studies); omega-3 fatty acids derived from alpha-linolenic acid were used (2 studies), or there were missing values (2 studies). The following reasons provided a rationale for the exclusion of additional studies (1 each): the authors did not use a randomized control trial (RCT); full access to the article was not possible; all participants were healthy, or aspirin was administered as well (S6 Text). Of the 16 studies eligible, 9 had published data on diabetic patients^29–37^, and 7 had published data on those with CVD^38–44^. The studies were published from 1990 to 2015 and were conducted throughout the world (six in the US, three in Iran, two in the UK, and one each in Australia, Spain, Finland, Israel, and China: S6 Text). Among these, lipid and inflammatory biomarkers investigated commonly included total cholesterol (11 studies), LDL and TG (10 studies each), and HDL (7 studies).

Overall, the findings from the studies included were considered to be at low risk of bias (S7 Text). Most studies used blind outcomes (patient reported), with no selective reporting (long-term > 6 weeks) or incomplete outcome data (16/16, 100%). Appropriate allocation concealment (9/16, 56.3%), generation of randomized sequences (4/16, 25%), and the blinding of participants and personnel (3/16, 18.8%) were clearly low in some studies (S7 Text).

### Diabetes

**Figure 2:**
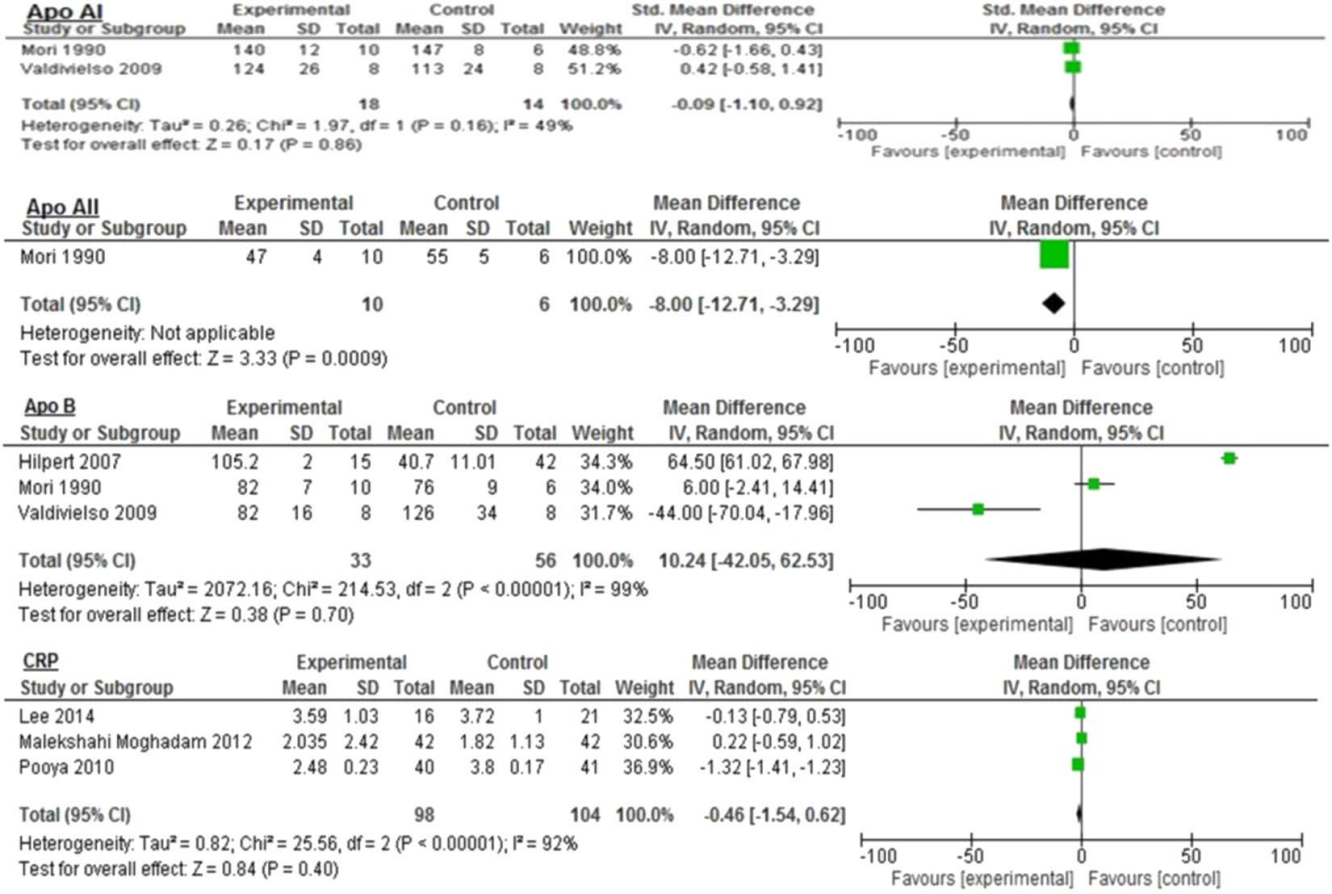
Impact of omega-3 fatty acids on Apo AI, Apo AII, Apo B, and CRP among diabetic patients.

**Figure 3:**
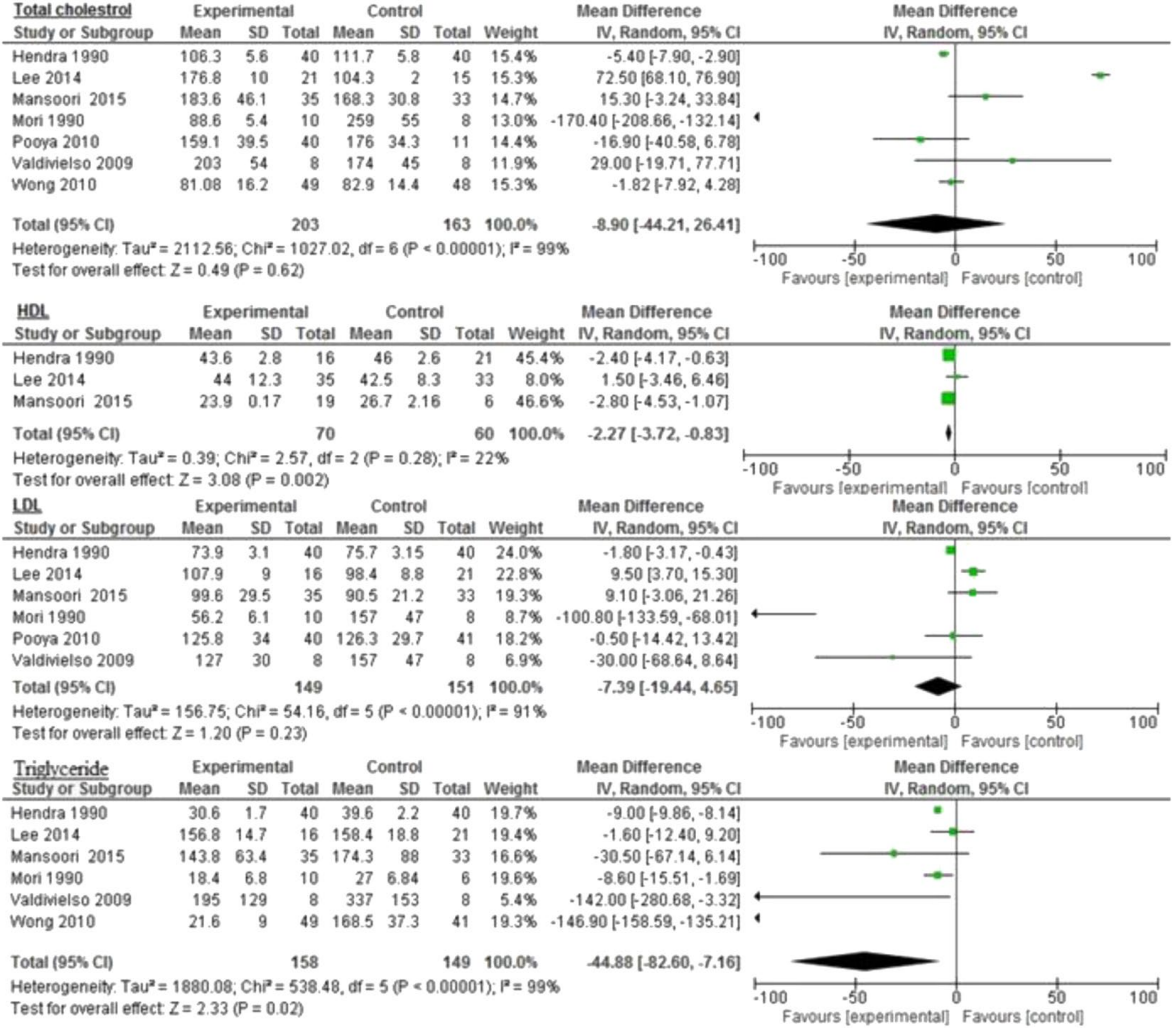
Impact of omega-3 fatty acids on total cholesterol, HDL, LDL, and triglycerides among diabetic patients.

**Figure 4:**
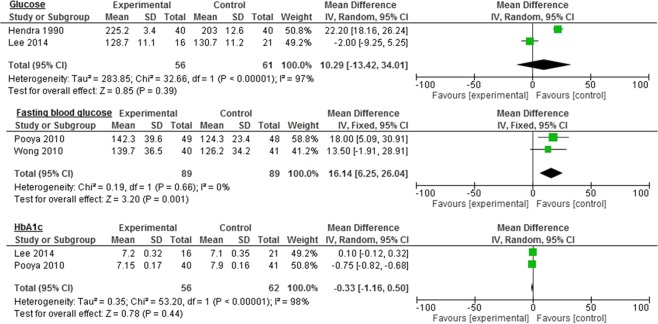
Impact of omega-3 fatty acids on glucose, fasting blood glucose, and HbA1c among diabetic patients.

Overall, the majority of inflammatory biomarkers potentially was associated with omega-3 intake compared to different control groups (Figs. 2, 3 and 4). Omega-3 fatty acids among diabetic patients were associated significantly with reduced Apo AII (−8.0 mg/dL 95% CI: −12.71, −3.29, *p* = 0.0009: Fig. 2); triglyceride (−44.88 mg/dL 95% CI: −82.6, −7.16, *p* < 0.0001, I^2^ = 99%), and HDL (−2.27 mg/dL 95% CI: −3.72, −0.83, *p* = 0.002; I^2^ = 22%: Fig. 3).

Similar reductions were seen in LDL (−7.39 mg/dL 95% CI: −19.44, 4.65, *p* = 0.23, I^2^ = 91%); total cholesterol (−8.9 mg/dL 95% CI: −44.21, 26.41, *p* = 0.62, I^2^ = 99%); HbA1c (−0.33 mg/dL 95% CI: −1.16, 0.5, *p* = 0.44, I^2^ = 98%); CRP (−0.46 mg/dL 95% CI: −1.54–0.62, *p* = 0.40, I^2^ = 92%), and Apo AI (−0.09 mg/dL 95% CI: −1.10–0.92, *p* = 0.86, I^2^ = 49%: Figs. 2–4). However, the levels were not statistically significant.

The control group demonstrated significantly improved fasting blood glucose levels compared to the omega-3 group (16.14 mg/dL 95% CI: 6.25, 26.04, *p* = 0.001, I^2^ = 0%) with no significant heterogeneity between the groups (Fig. 4). Similar, but nonsignificant, results were seen in glucose (10.29 mg/dL 95% CI: −13.42, 34.01, *p* = 0.39, I^2^ = 97%) and Apo B levels (10.24 mg/dL 95% CI: −42.05–62.53, *p* = 0.70, I^2^ = 99%: Figs. 2 and 4).

### Cardiovascular disease

**Figure 5:**
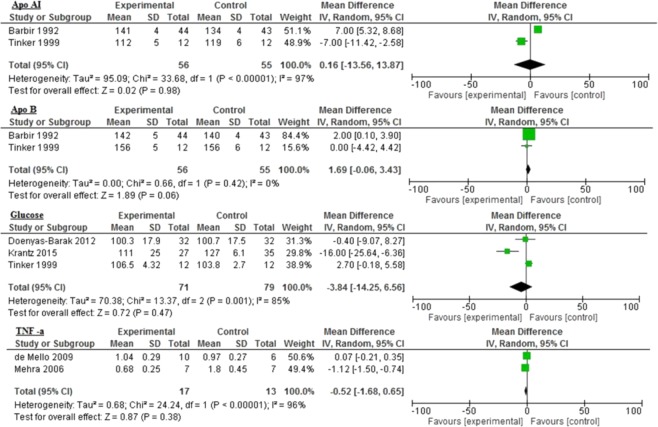
Impact of omega-3 fatty acids on Apo AI, Apo B, glucose, and TNF-α among CVD patients.

**Figure 6:**
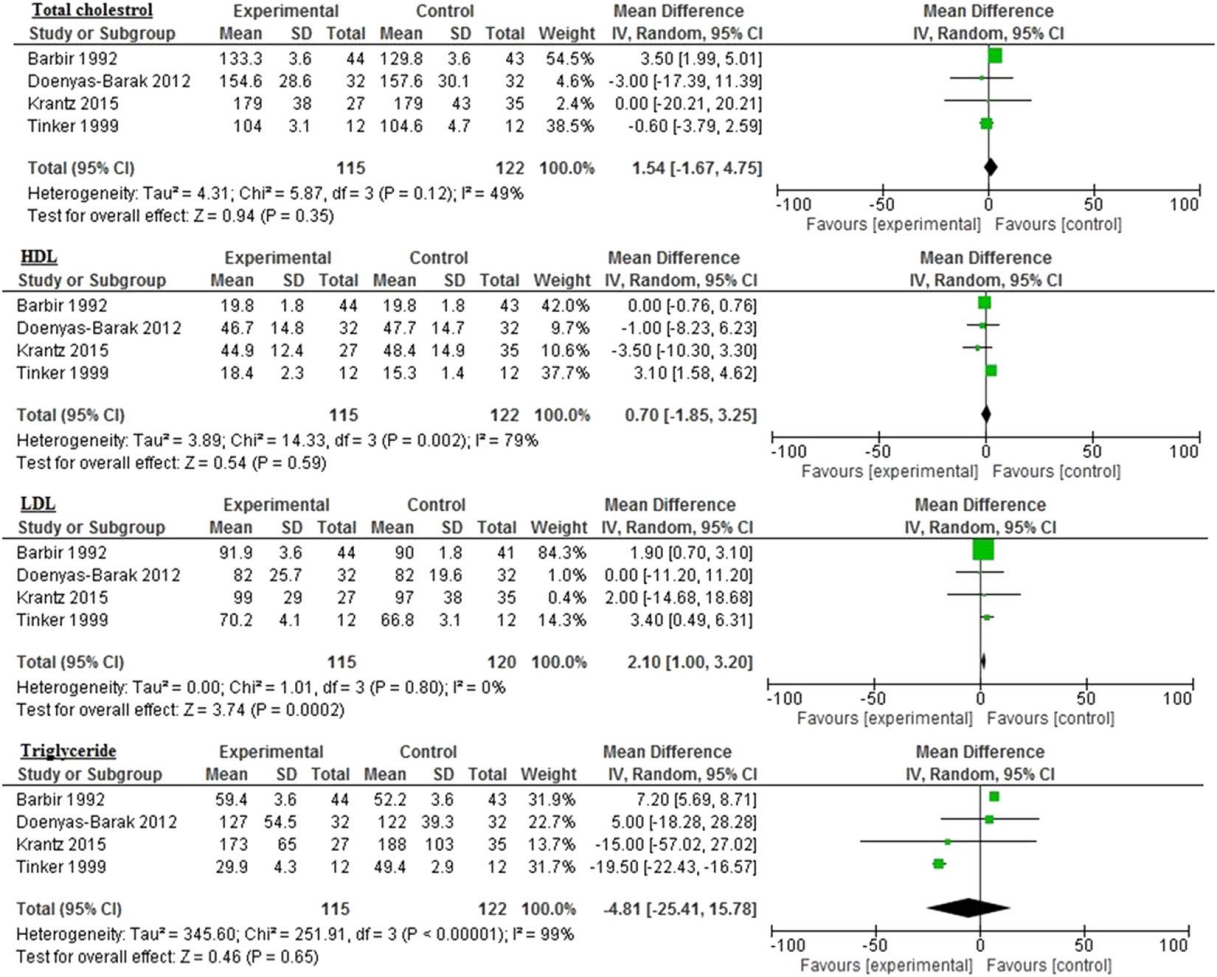
Impact of omega-3 fatty acids on total cholesterol, HDL, LDL, and triglycerides among CVD patients.

The reductions in inflammatory biomarkers varied with different controls. There was a statistically significant increase in LDL levels in the control group (2.10 mg/dL 95% CI: 1.00, 3.20, *p* = 0.0002, I^2^ = 0%) compared to the omega-3 group (Fig. 5). While the control group demonstrated improved levels of TG (−4.81 mg/dL 95% CI: −25.41, 15.78, *p* = 0.65, I^2^ = 99%), glucose (−3.84 mg/dL 95% CI: −14.25, 6.56; *p* = 0.47; I^2^ = 85%), and TNF-α (−0.52 mg/dL 95% CI: −1.68, 0.65, *p* = 0.38, I^2^ = 96%), the improvement was not statistically significant (Figs. 5 and 6).

Compared to different control groups, omega-3 polyunsaturated fatty acids seem to be associated with reductions in the following inflammatory biomarkers: Apo B (1.69 mg/dL 95% CI: −0.06, 3.43, *p* = 0.06, I^2^ = 0%); Apo AI (0.16 mg/dL 95% CI: −13.56, 13.87, *p = *0.98, I^2^ = 97%); total cholesterol (1.54 mg/dL 95% CI: −1.67, 4.75, *p* = 0.35, I^2^ = 49%), and HDL (0.70 mg/dL 95% CI: −1.85, 3.25, *p* = 0.59, I2 = 79%: Figs. 5 and 6).

### Sensitivity analysis

When we detected substantial heterogeneity, we planned to conduct a subgroup and sensitivity analysis to explore the possible explanations of the results. Although there were insufficient data for us to explore subgroups in this review, we planned sensitivity analyses for the inflammatory and lipid outcomes to determine whether the conclusions were sufficiently strong for the decisions made with respect to eligibility and analysis. We considered whether the review conclusions would have changed if (1) the eligibility was restricted to at least four weeks of follow up, or (2) a specific control group was used. However, because of insufficient data, we were unable to perform sensitivity analyses in this review, and the quality of the evidence of bias was low overall.

## Discussion

Our results indicated that, compared to different control groups, omega-3 polyunsaturated fatty acids are associated statistically significantly with reduced levels of Apo AII, triglycerides, and HDL, as well as improved levels of fasting blood glucose among diabetic patients. Omega-3 fatty acids also are associated with statistically significant increases in LDL levels among CVD patients. Other biomarkers may experience beneficial reductions with omega-3 use among diabetic or cardiovascular patients.

The ultimate target of any inflammatory process is to clear the insults and leukocytes from lesions and resolve and restore tissue homeostasis^45^. The restoration of tissue homeostasis always begins with inflammatory lipid mediators (e.g., leukotrienes). The concept of inflammation resolution depends primarily on the active class switch in the mediators from classical prostaglandins and leukotrienes to promising newer immunoresolvents molecules^1^.

Endogenous immunoresolvent lipid mediator molecules (e.g., resolvins, protectins, lipoxins, and maresins) are produced during the inflammation resolution phase and have anti-inflammatory and pro-resolving functions, which have promising findings that may treat several human diseases^46^. Although lipoxins are derived from different sources than are others (arachidonic acid vs. dietary fatty acids, primarily fish oil), together, they all can help inhibit neutrophil recruitment, promote tissue regeneration and the lymphatic removal of phagocytes, and attenuate proinflammatory gene expression^47–52^.

Several strategies have been used to control diabetes, such as lifestyle changes and eating plans (e.g., low-glycemic index diet, exercise)^53–55^, and medications such as oral hypoglycemic agents^56^ or insulin^57^. Some studies have introduced omega-3 fatty acids to decrease levels of fasting plasma glucose (FPG) and improve lipid profiles, inflammatory mediators, and reduce insulin resistance^58–60^. However, low dosages of omega-3 fatty acids may have limited effects on insulin resistance, inflammatory markers, and lipid profiles among HIV patients and certain other populations^61^.

These molecules’ precise mechanisms of action in diabetic or CVD patients are not yet clear. However, omega-3 fatty acids may affect insulin metabolism and lipid profiles in the following four ways: reducing LDL/cholesterol synthesis; enhancing lipid profiles and receptor activity in the liver (e.g., affecting LDL receptors, increasing LDL/cholesterol catabolism)^62,63^; improving insulin function and glucose tolerance^58,60,64^, and increasing the expression of AMP-activated protein kinase (AMPK)^65^. Several hypotheses have been proposed to explain omega-3′s effect, including elevated adiponectin levels, the inhibition of proinflammatory cytokines, and nuclear factor-kB (NF-κB) protein expression. These molecules can have anti-diabetic properties because of improved insulin metabolism and the anti-atherosclerotic and anti-inflammatory effects attributable to the resolution of inflammation, as mentioned previously^66^. As a consequence, the reduced proinflammatory mediators on the one hand, and the increased production of anti-inflammatory molecules, such as adiponectin, on the other will improve insulin resistance^46,64,65^.

Moreover, high dietary intake of omega-3 fatty acids may be associated with low inflammation and endothelial function in patients with hypercholesterolemia^62,67^, although omega-3 fatty acids’ effects on those with CVD are unclear. However, omega-3 intake may change the HDL cholesterol subfraction composition and absolute size. Omega-3 fatty acids also lower triglycerides by reducing the hepatic secretion of VLDL cholesterol^68^. Further, it may decrease triglycerides and increase LDL cholesterol in patients with hypertriglyceridemia^69^. Our study has shown LDL level increases and triglyceride level reductions. However, this mechanism remains unclear and some studies have shown neutral effects^70^.

Thies *et al*. suggested that another way in which omega-3 fatty acids might act on CVD patients is by stabilizing advanced atherosclerotic plaques and reducing their anti-inflammatory effects thereby^71^. Few studies have examined omega-3 fatty acids’ effects on TNF-α levels and the results are inconsistent^72–75^. However, this study showed that omega-3 fatty acids improved TNF-alpha levels.

Very few randomized controlled trials have investigated omega-3 intake’s potential effects on inflammatory biomarkers, insulin metabolism, and lipid mediators in diabetic and CVD patients^59,67,70,71,76–78^. Thus, this systematic review summarized all published results of omega-3 fatty acid intake on these aspects. Perhaps omega-3 fatty acids’ use may be a new approach in the future to improve lipid profiles and reduce inflammatory biomarkers, which currently is a popular topic among those in the medical community. Several investigators have proposed this hypothesis because of their beneficial effects on morphologic and inflammatory markers^76,77^. However, until long-term follow-up studies are conducted, the results must be interpreted with caution.

Our review has several strengths and limitations. Its strengths include our comprehensive search strategy, the inclusion only of randomized clinical trials, the low risk of bias among the studies selected, and restricted inclusion criteria (e.g., omega-3 intake of more than 1000 mg/day limited to EPA and DHA sources). These criteria allowed us to investigate their potential benefits on lipid profiles and inflammatory biomarkers systematically. The limitations pertain primarily to the inability to draw strong conclusions based on significant statistical and clinical results. This outcome may be attributed to one or more of the following: different populations/study samples and designs, a lack of dependent variable baseline measurements in some studies, the possibility of lifestyle interactions, differences in the severity of study participants’ chronic disease, variation in omega-3 fatty acid dosages, or the studies’ duration.

## Conclusion

Omega-3 polyunsaturated fatty acids may be associated with improvements in inflammatory biomarkers and lipid profiles among diabetic and cardiovascular patients. However, the review did not identify clear benefits for these markers and profiles. Clinicians should be aware of these potential benefits before prescribing omega-3, and it is essential that patients consult with clinicians before any omega-3 intake because of the current limited data on its effects.

## Supplementary information

S1 PRISMA 2009 checklist

S2 Text Protocol

S3 Text Search stratgy

S4 Text Risk of bias

S5 Text funnal plot

S6 List of excluded articles and reason of exclusion

S7 Studies chractristics
