# Supplementary material for: Omega-3 Fatty Acids Effects on Inflammatory Biomarkers and Lipid Profiles among Diabetic and Cardiovascular Disease Patients: A Systematic Review and Meta-Analysis
Source: Sci Rep. 2019 Dec 11;9:18867. doi: 10.1038/s41598-019-54535-x (PMC6906408; doi:10.1038/s41598-019-54535-x)
Supplement: Supplementary file 2 — S2 Text Protocol [file 41598_2019_54535_MOESM2_ESM.docx]

**S2: Protocol**

**Omega-3 Fatty Acids Effects on Inflammatory Biomarkers and Lipid Profiles among Diabetic and Cardiovascular Disease Patients: A Systematic Review and Meta-Analysis**

*Zuhair S. Natto BDS, MPH, MSc, DrPH, Wael Yaghmoor*  *BDS, MSc , Heba K. Alshaeri PharmD, MPH, PhD & Thomas E. Van Dyke DDS, MS, PhD.*

**The effects of omega-3 fatty acids on inflammatory biomarkers among diabetic and cardiovascular disease patients: a systematic review and meta-analysis of randomized clinical trials**

Zuhair Natto, Wael Yaghmoor, Heba Alshaeri, Thomas Van Dyke

**Citation**

Zuhair Natto, Wael Yaghmoor, Heba Alshaeri, Thomas Van Dyke. The effects of omega-3 fatty acids on inflammatory biomarkers among diabetic and cardiovascular disease patients: a systematic review and meta-analysis of randomized clinical trials. PROSPERO 2018 CRD42018098744 Available from: <http://www.crd.york.ac.uk/PROSPERO/display_record.php?ID=CRD42018098744>

**Review question**

What are the effects of omega-3 fatty acids on inflammatory biomarkers among diabetic and cardiovascular disease patients?

PICOS:

P (Population): diabetic or cardiovascular disease patients;

I (Intervention): any form of omega-3:

C (Comparisons): any;

O (Outcomes): inflammatory biomarkers;

S (Study design): randomized clinical trials.

**Searches**

The following databases will be searched:

1- MEDLINE;

2- Embase;

3- Scopus;

4- Web of Science;

5- The Cochrane Central Register of Controlled Trials (CENTRAL).

In addition, internet search engines such as Google Scholar will be screened for relevant literature, and a hand search of the references of included articles will also be undertaken.

Additional search strategy information can be found in the attached PDF document (link provided below).

**Types of study to be included**

Randomized controlled trials will be included to assess the effects of omega-3 fatty acids.

Studies will included if they report changes in inflammatory biomarkers such as LDL, HDL , total cholesterol, TG, HbAIC, Apo AII, CRP, TNF-a, and their SDs have been included, or data with which to calculate these values.

**Condition or domain being studied**

Omega-3 fatty acids, diabetes, cardiovascular disease.

**Participants/population**

Patients with diabetes or cardiovascular disease.

Inclusion:

1- Humans;

2- With an intake of at least 1000mg of omega-3.

Exclusion:

1- Healthy participants or risk factors studies;

2- Any omega-3 derived from alpha-linolenic acid (ALA).

**Intervention(s), exposure(s)**

Omega-3 or EPA (eicosapentaenoic acid) and DHA (docosahexaenoic acid), or one of the following lipid mediators: lipoxins (lipoxin A4, lipoxin B4), resolvins (resolvin E1, resolvin E2, resolvin D1), protectin (protectin D1, AT-PD1), or maresin (maresin 1).

**Comparator(s)/control**

Any placebo control, or a comparison arm or diet.

**Context**

**Main outcome(s)**

Changes in LDL, HDL, total cholesterol, TG, HbAIC, Apo AII, CRP, TNF-a, and their SDs, or data with which to calculate these values.

**Additional outcome(s)**

None.

**Data extraction (selection and coding)**

Data extraction will be conducted by two independent authors (ZN and WY) and any discrepancies arising will be resolved through discussion.

The following information from each study will be extracted: country, study type, lipid mediators, area, total sample size, gender, age, duration, outcome, population, test or control type, dose, sample size, gender, age, mean and SD before and after, or mean and SD difference.

**Risk of bias (quality) assessment**

The quality of the included studies will be assessed using the Cochrane Collaboration’s tool for the assessment of risk of bias by two independent authors (ZN and WY). Any discrepancies arising will be resolved through discussion.

**Strategy for data synthesis**

We will use mean changes in inflammatory biomarker levels for all groups, such as LDL, HDL, total cholesterol, TG, HbAIC, Apo AII, CRP, TNF-a, and their SDs, or data with which to calculate these values.

For studies that do not provide SDs, a correlation coefficient of 0.5 will be considered for the missing SDs, based on method of Follmann et al.

For each meta-analysis, mean differences (MDs) for continuous outcomes will be applied, combined across studies by random-effects, and all units of measurements will be converted to a standard single unit (mg/dl).

The results will be summarized using forest plots, using the I² (I²) statistic for heterogeneity. A sensitivity analysis will also be carried out to explore the extent to which inferences might depend on a particular duration, study or a number of publications.

Publication bias will discovered by looking over Begg’s funnel plots.

**Analysis of subgroups or subsets**

If the necessary data are available, subgroup analyses will be carried out based on age, gender, doses and duration.

**Contact details for further information**

Zuhair Natto

z_world@hotmail.com

**Organisational affiliation of the review**

King Abdulaziz University

**Review team members and their organisational affiliations**

Dr Zuhair Natto. King Abdulaziz UniversityDr Wael Yaghmoor. Boston UniversityDr Heba Alshaeri. MCPHS UniversityProfessor Thomas Van Dyke. Harvard University

**Anticipated or actual start date**

26 May 2018

**Anticipated completion date**

31 May 2019

**Funding sources/sponsors**

None

**Conflicts of interest**

None specified.

**Language**

(there is not an English language summary)

**Country**

Saudi Arabia

**Stage of review**

Review_Ongoing

**Subject index terms status**

Subject indexing assigned by CRD

**Subject index terms**

Biomarkers; Cardiovascular Diseases; Diabetes Mellitus; Dietary Supplements; Fatty Acids, Omega-3; Functional Food; Humans; Inflammation; Nutrition Therapy
