# Supplementary material for: Omega-3 Fatty Acids Effects on Inflammatory Biomarkers and Lipid Profiles among Diabetic and Cardiovascular Disease Patients: A Systematic Review and Meta-Analysis
Source: Sci Rep. 2019 Dec 11;9:18867. doi: 10.1038/s41598-019-54535-x (PMC6906408; doi:10.1038/s41598-019-54535-x)
Supplement: Supplementary file 3 — S3 Text Search stratgy [file 41598_2019_54535_MOESM3_ESM.docx]

**S3: Search Strategy**

**Omega-3 Fatty Acids Effects on Inflammatory Biomarkers and Lipid Profiles among Diabetic and Cardiovascular Disease Patients: A Systematic Review and Meta-Analysis**

*Zuhair S. Natto BDS, MPH, MSc, DrPH, Wael Yaghmoor*  *BDS, MSc , Heba K. Alshaeri PharmD, MPH, PhD & Thomas E. Van Dyke DDS, MS, PhD.*

Database: Ovid MEDLINE(R) 1946 to May Week 3 2018

1 exp Fish Oils/ or exp Fatty Acids, Omega-3/

2 exp Diabetes Mellitus, Experimental/ or exp Diabetes Mellitus/ or exp Diabetes Mellitus, Type 2/ or exp Diabetes Mellitus, Type 1/

3 exp Coronary Disease/ or exp Cardiovascular Diseases/ or exp Cerebrovascular Disorders/

4 1 and 2

5 1 and 3

6 exp Fish Oils/ and exp Fatty Acids, Omega-3/

7 exp Eicosapentaenoic Acid/

8 exp Docosahexaenoic Acids/

9 7 and 8

10 7 or 8

11 2 and 9 and 10

12 3 and 9 and 10

13 lipoxin.mp. or exp Lipoxins/

14 Resolvin.mp.

15 Protectin.mp.

16 Maresin.mp.

17 2 and 13

18 2 and 14

19 2 and 15

20 2 and 16

21 3 and 13

22 3 and 14

23 3 and 15

24 3 and 16

25 diabetes$.mp.

26 6 and 25

27 9 and 25

28 10 and 25

29 13 and 25

30 14 and 25

31 15 and 25

32 16 and 25

Database: Embase Classic+Embase 1947

1 fish oils/ or fatty acids/ or omega-3/

2 Diabetes Mellitus/ or Type 2/ or Type 1/

3 Coronary Disease/ Cardiovascular Diseases/ or Cerebrovascular Disorders/

4 or/1-2

5 or/1-3

6 exp humans / not animal/

7 lipoxin/

8 Resolvin/.

9 Protectin/

10 Maresin/

11 or/6-10

12 4 and 11

13 5 and 11

14 clinical trial/ or multicenter study/ or phase 1 clinical trial/ or phase 2 clinical trial/ or phase 3 clinical trial/ or phase 4 clinical trial/

15 12 and 14

16 13 and 14

Database: Cochrane Central Register of Controlled Trials [Ovid]

1 fish oils/ or fatty acids/ or omega-3/

2 Diabetes Mellitus/ or Type 2/ or Type 1/

3 Coronary Disease/ Cardiovascular Diseases/ or Cerebrovascular Disorders/

4 or/1-2

5 or/1-3

6 exp humans / not animal/

7 lipoxin/

8 Resolvin/.

9 Protectin/

10 Maresin/

11 or/6-10

12 4 and 11

13 5 and 11

14 clinical trial/ or multicenter study/ or phase 1 clinical trial/ or phase 2 clinical trial/ or phase 3 clinical trial/ or phase 4 clinical trial/

15 12 and 14

16 13 and 14

Database: Web of Science [Thomson Reuters]

Science Citation Index Expanded (SCI-EXPANDED) --1900-present

Social Sciences Citation Index (SSCI) --1956-present

Conference Proceedings Citation Index- Science (CPCI-S) --1990-present

Conference Proceedings Citation Index- Social Science & Humanities (CPCI-SSH) --1990-present

Set

# 9 #7 OR #6 OR #4

Indexes=SCI-EXPANDED, SSCI, CPCI-S, CPCI-SSH Timespan=All years

# 8 #7 OR #6 OR #5

Indexes=SCI-EXPANDED, SSCI, CPCI-S, CPCI-SSH Timespan=All years

# 7 TS=("controlled clinical comparison" or "controlled clinical experiment" or "controlled clinical study" or "controlled clinical test" or "control* trial" or "control* group" or "control* study")

Indexes=SCI-EXPANDED, SSCI, CPCI-S, CPCI-SSH Timespan=All years

# 6 TS=("clinical trial*" or "clinical drug trial*" or "controlled trial*")

Indexes=SCI-EXPANDED, SSCI, CPCI-S, CPCI-SSH Timespan=All years

# 5 #2 OR #1

Indexes=SCI-EXPANDED, SSCI, CPCI-S, CPCI-SSH Timespan=All years

# 4 #3 OR #1

Indexes=SCI-EXPANDED, SSCI, CPCI-S, CPCI-SSH Timespan=All years

# 3 TS=( (coronary Disease or cardiovascular diseases or cerebrovascular disorders))

Indexes=SCI-EXPANDED, SSCI, CPCI-S, CPCI-SSH Timespan=All years

# 2 TS=(( diabetes Mellitus or type 2 or Type 1)

Indexes=SCI-EXPANDED, SSCI, CPCI-S, CPCI-SSH Timespan=All years

# 1 TS=(( fish oils or fatty acids or omega-3)

Indexes=SCI-EXPANDED, SSCI, CPCI-S, CPCI-SSH Timespan=All years
