# Supplementary material for: Omega-3 Fatty Acids Effects on Inflammatory Biomarkers and Lipid Profiles among Diabetic and Cardiovascular Disease Patients: A Systematic Review and Meta-Analysis
Source: Sci Rep. 2019 Dec 11;9:18867. doi: 10.1038/s41598-019-54535-x (PMC6906408; doi:10.1038/s41598-019-54535-x)
Supplement: Supplementary file 4 — S4 Text Risk of bias [file 41598_2019_54535_MOESM4_ESM.docx]

**S4: Risk of bias**

**Omega-3 Fatty Acids Effects on Inflammatory Biomarkers and Lipid Profiles among Diabetic and Cardiovascular Disease Patients: A Systematic Review and Meta-Analysis**

*Zuhair S. Natto BDS, MPH, MSc, DrPH, Wael Yaghmoor*  *BDS, MSc , Heba K. Alshaeri PharmD, MPH, PhD & Thomas E. Van Dyke DDS, MS, PhD.*

Table 1: The Cochrane Collaboration's tool for assessing risk of bias for the Included Studies DM

| Study | Random sequence | Allocation concealment | Blinding of participants and personnel | Blinding of outcome (patient reported) | Incomplete outcome data (long-term (>6 weeks) | Selective reporting | Other bias |
| --- | --- | --- | --- | --- | --- | --- | --- |
| Mansoori A et al. 2015 | Low | Low | Unclear | Low | Low | Low | Low |
| Malekshahi Moghadam A et al. 2012 | Unclear | Low | Unclear | Low | Low | Low | Low |
| Lee TC et al. 2014 | Unclear | Low | Low | Low | Low | Low | Low |
| Valdivielso P et al. 2009 | High | High | High | Low | Low | Low | Low |
| Hilpert KF et al. 2007 | Unclear | Unclear | Unclear | Low | Low | Low | Low |
| Hendra TJ et al. 1990 | Unclear | Unclear | Unclear | Low | Low | Low | High |
| Mori TA et al. 1990 | High | High | High | Low | Low | Low | Low |
| Pooya SH et al. 2010 | Unclear | Low | Unclear | Low | Low | Low | Low |
| Wong CY et al. 2010 | Low | Low | Low | Low | Low | Low | Low |
| Krantz MJ et al. 2015 | Unclear | Low | Unclear | Low | Low | Low | Low |
| Root M et al. 2013 | Low | Unclear | Unclear | Low | Low | Low | High |
| Tinker LF et al. 1999 | Unclear | Unclear | Unclear | Low | Low | Low | Low |
| Mehra MR et al. 2006 | Unclear | Low | Unclear | Low | Low | Low | Low |
| Barbir M et al. 1992 | Unclear | Low | Low | Low | Low | Low | Low |
| de Mello VD et al. 2009 | Low | Low | Unclear | Low | Low | Low | Low |
| Doenyas-Barak K et al. 2012 | NA | NA | Unclear | Low | Low | Low | Low |
