# Supplementary material for: Omega-3 Fatty Acids Effects on Inflammatory Biomarkers and Lipid Profiles among Diabetic and Cardiovascular Disease Patients: A Systematic Review and Meta-Analysis
Source: Sci Rep. 2019 Dec 11;9:18867. doi: 10.1038/s41598-019-54535-x (PMC6906408; doi:10.1038/s41598-019-54535-x)
Supplement: Supplementary file 5 — S5 Text funnal plot [file 41598_2019_54535_MOESM5_ESM.docx]

**S5: Funnel plot**

**Omega-3 Fatty Acids Effects on Inflammatory Biomarkers and Lipid Profiles among Diabetic and Cardiovascular Disease Patients: A Systematic Review and Meta-Analysis**

*Zuhair S. Natto BDS, MPH, MSc, DrPH, Wael Yaghmoor*  *BDS, MSc , Heba K. Alshaeri PharmD, MPH, PhD & Thomas E. Van Dyke DDS, MS, PhD.*

Risk of publication bias summary:

**
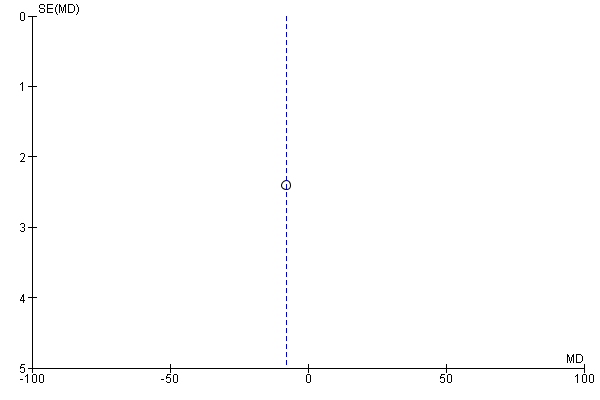
Caption**

Funnel plot of comparison: DM, outcome: Apo A II.


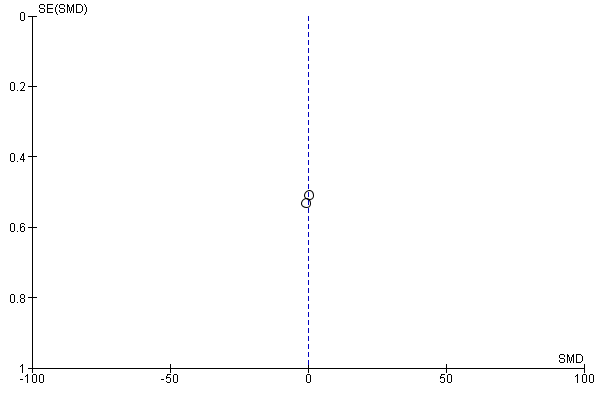


Funnel plot of comparison: DM, outcome: Apo A I.

Forest plot of comparison: 7 DM, outcome: 7.3 Apo B.


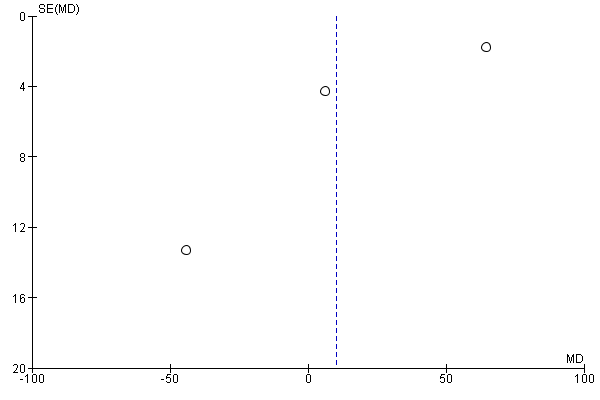


Funnel plot of comparison: DM, outcome: Apo B.


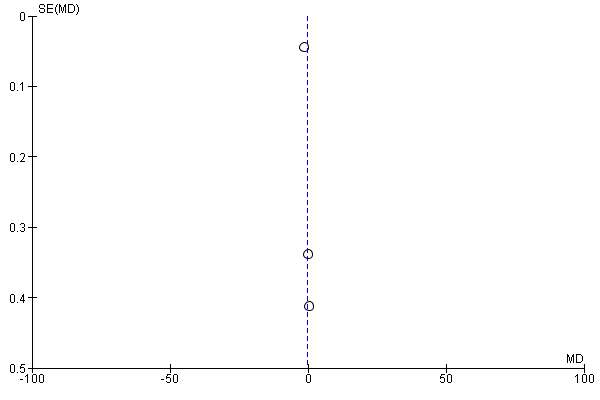


Funnel plot of comparison: DM, outcome: CRP.

**
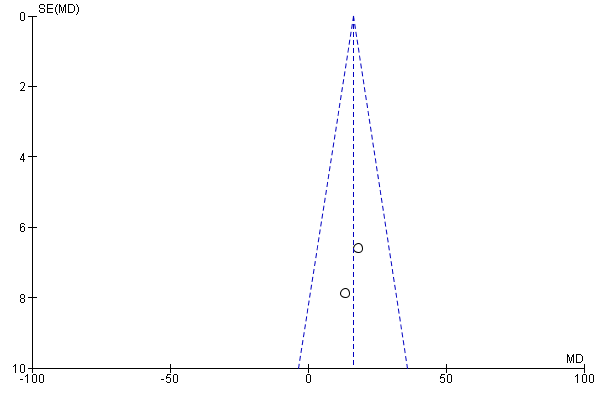
**

Funnel plot of comparison: DM, outcome: Fasting blood glucose.


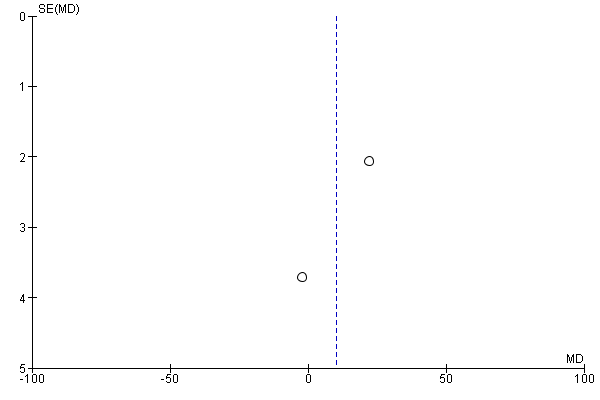


Funnel plot of comparison: DM, outcome: Glucose.


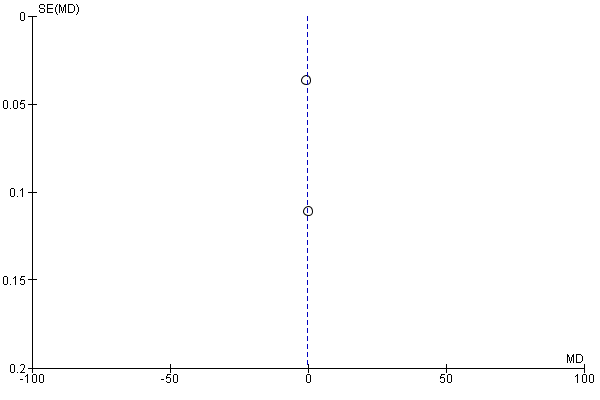


Funnel plot of comparison: DM, outcome: HbAIc.


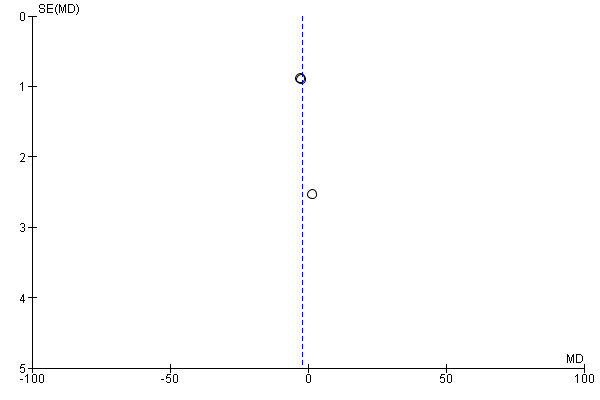


Funnel plot of comparison: DM, outcome: HDL.


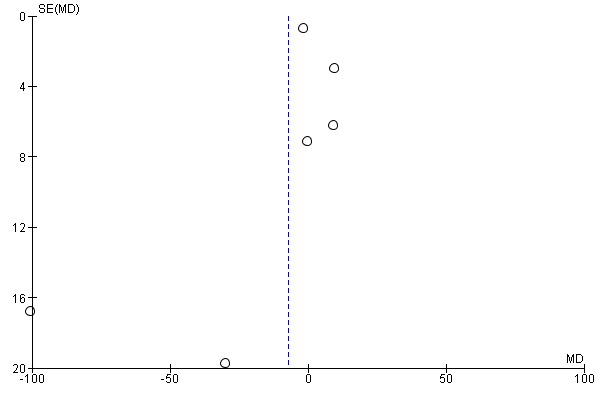


Funnel plot of comparison: DM, outcome: 7. LDL.


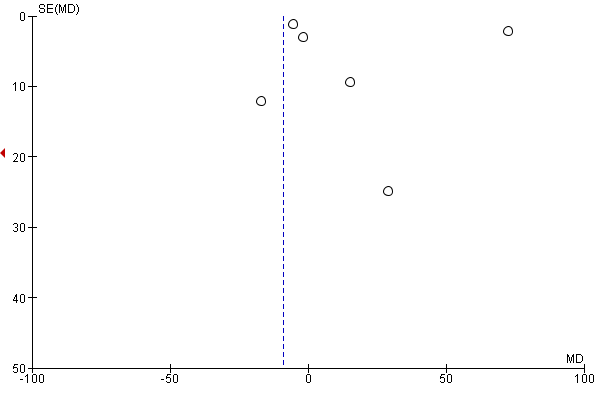


Funnel plot of comparison: DM, outcome: Total cholestrol.


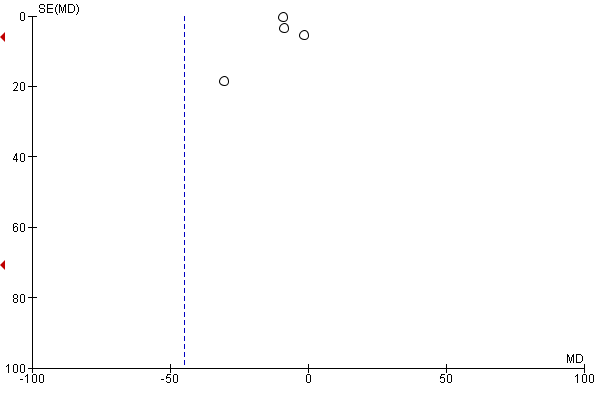


Funnel plot of comparison: DM, outcome: TG.


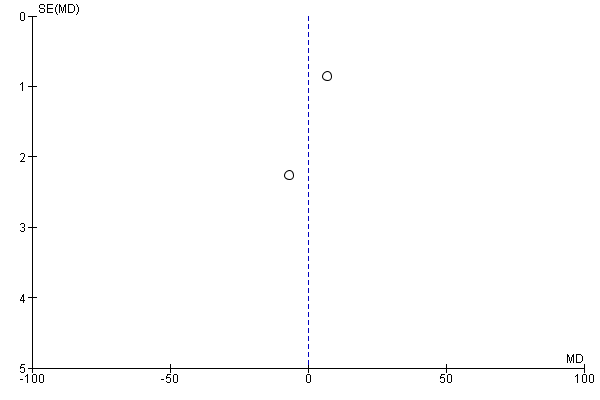


Funnel plot of comparison: CVD, outcome: Apo A.


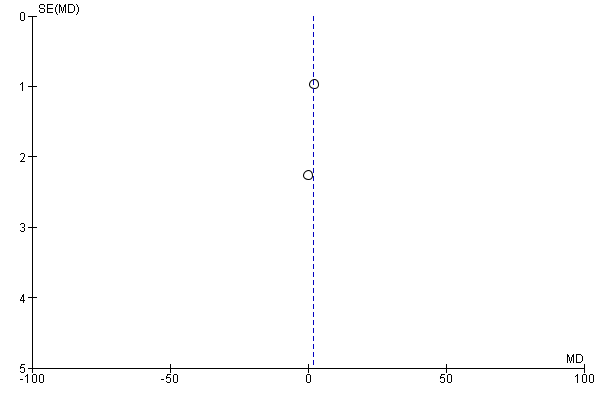


Funnel plot of comparison: CVD, outcome: Apo B.


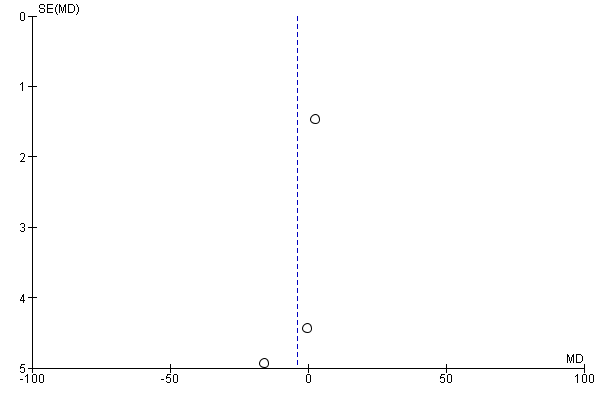


Funnel plot of comparison: CVD, outcome: Glucose.


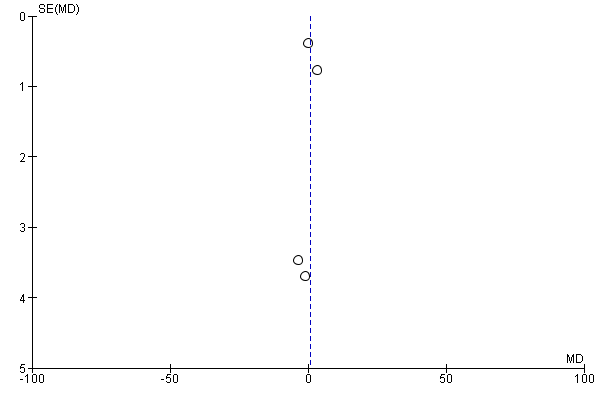


Funnel plot of comparison: CVD, outcome: HDL.


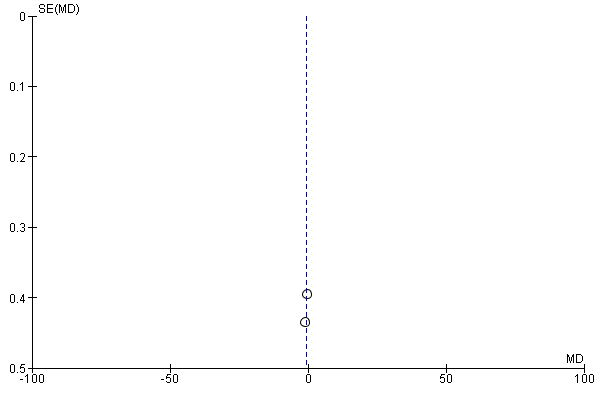


Funnel plot of comparison: CVD, outcome: Hemoglobin.


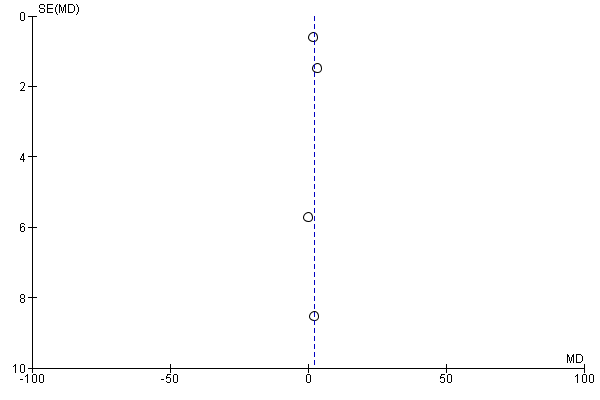


Funnel plot of comparison: CVD, outcome: LDL.


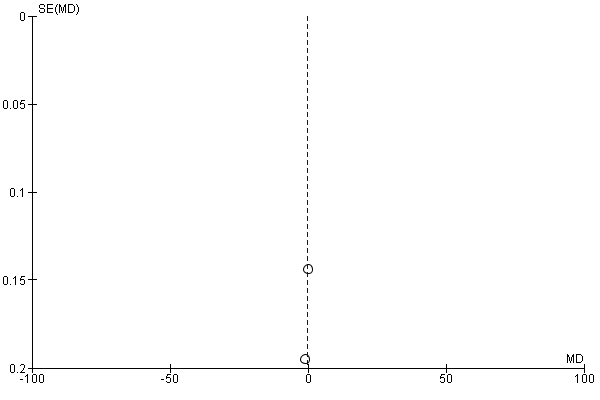


Funnel plot of comparison: CVD, outcome: TNF-a.


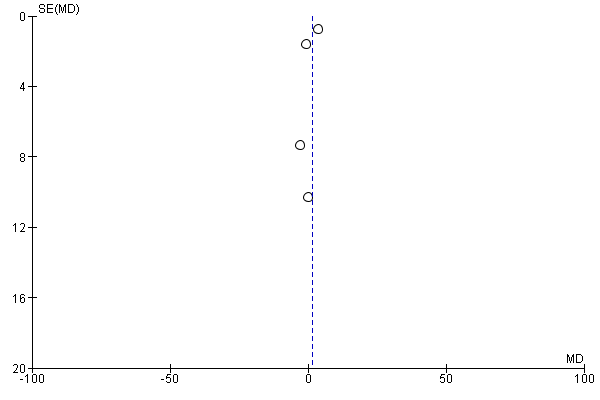


Funnel plot of comparison: CVD, outcome: Total Cholesterol.


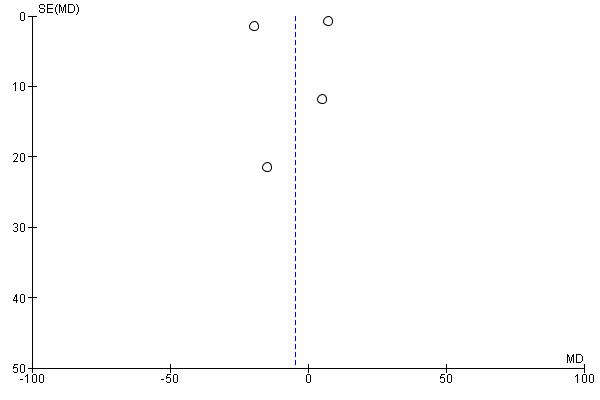


Funnel plot of comparison: CVD, outcome: TG.
