# Supplementary material for: Omega-3 Fatty Acids Effects on Inflammatory Biomarkers and Lipid Profiles among Diabetic and Cardiovascular Disease Patients: A Systematic Review and Meta-Analysis
Source: Sci Rep. 2019 Dec 11;9:18867. doi: 10.1038/s41598-019-54535-x (PMC6906408; doi:10.1038/s41598-019-54535-x)
Supplement: Supplementary file 6 — S6 List of excluded articles and reason of exclusion [file 41598_2019_54535_MOESM6_ESM.docx]

**S6: List of excluded articles and reasons of exclusion**

**Omega-3 Fatty Acids Effects on Inflammatory Biomarkers and Lipid Profiles among Diabetic and Cardiovascular Disease Patients: A Systematic Review and Meta-Analysis**

*Zuhair S. Natto BDS, MPH, MSc, DrPH, Wael Yaghmoor*  *BDS, MSc , Heba K. Alshaeri PharmD, MPH, PhD & Thomas E. Van Dyke DDS, MS, PhD.*

**List of full text excluded articles and reason of exclusion:**

| **Reference** | **Reason for exclusion** |
| --- | --- |
| Merino J, Sala-Vila A, Kones R, Ferre R, Plana N, Girona J, Ibarretxe D, Heras M, Ros E, Masana L. Increasing long-chain n-3PUFA consumption improves small peripheral artery function in patients at intermediate-high cardiovascular risk. J Nutr Biochem. 2014 Jun;25(6):642-6. | omega-3 dosage in tertile and can not determined |
| Dawczynski C, Massey KA, Ness C, Kiehntopf M, Stepanow S, Platzer M, GrÃ¼n M, Nicolaou A, Jahreis G. Randomized placebo-controlled intervention with n-3 LC-PUFA-supplemented yoghurt: effects on circulating eicosanoids and cardiovascular risk factors. Clin Nutr. 2013 Oct;32(5):686-96. | omega-3 < 1g per day |
| Andric T, Weiss TW, Huber K, Arnesen H, Seljeflot I. Effects of diet and/or n-3 fatty acid supplementation on components of the interleukin-6 trans-signalling system in elderly men. Scand J Clin Lab Invest. 2015;75(8):646-51. | used median in the unit |
| Nigam A, Talajic M, Roy D, Nattel S, Lambert J, Nozza A, Jones P, Ramprasath VR, O'Hara G, Kopecky S, Brophy JM, Tardif JC; AFFORD Investigators. Fish oil for the reduction of atrial fibrillation recurrence, inflammation, and oxidative stress. J Am Coll Cardiol. 2014 Oct 7;64(14):1441-8. | no change or before values |
| Hoogeveen EK, Geleijnse JM, Kromhout D, van't Sant P, Gemen EF, Kusters R, Giltay EJ. No effect of n-3 fatty acids supplementation on NT-proBNP after myocardial infarction: the Alpha Omega Trial. Eur J Prev Cardiol. 2015 May;22(5):648-55. | no full article |
| VeljoviÄ M, PopadiÄ A, VukiÄ Z, IliÄ R, TrifunoviÄ Z, AntunoviÄ M, MandariÄ V, Tisma S, MarkoviÄ Z. Myocardial protection during elective coronary artery bypasses grafting by pretreatment with omega-3 polyunsaturated fatty acids. Vojnosanit Pregl. 2013 May;70(5):484-92. | no inflammatory biomarkers |
| Masson S, Marchioli R, Mozaffarian D, Bernasconi R, Milani V, Dragani L, Tacconi M, Marfisi RM, Borgese L, Cirrincione V, Febo O, Nicolis E, Maggioni AP, Tognoni G, Tavazzi L, Latini R. Plasma n-3 polyunsaturated fatty acids in chronic heart failure in the GISSI-Heart Failure Trial: relation with fish intake, circulating biomarkers, and mortality. Am Heart J. 2013 Feb;165(2):208-15.e4. | No numbers |
| Foroughinia F, Salamzadeh J, Namazi MH. Protection from procedural myocardial injury by omega-3 polyunsaturated fatty acids (PUFAs): is related with lower levels of creatine kinase-MB (CK-MB) and troponin I?. Cardiovasc Ther. 2013 Oct;31(5):268-73 | used median in the unit |
| Ballantyne CM, Bays HE, Kastelein JJ, Stein E, Isaacsohn JL, Braeckman RA, Soni PN. Efficacy and safety of eicosapentaenoic acid ethyl ester (AMR101) therapy in statin-treated patients with persistent high triglycerides (from the ANCHOR study). Am J Cardiol. 2012 Oct 1;110(7):984-92. | used median in the unit |
| Mackay I, Ford I, Thies F, Fielding S, Bachoo P, Brittenden J. Effect of Omega-3 fatty acid supplementation on markers of platelet and endothelial function in patients with peripheral arterial disease. Atherosclerosis. 2012 Apr;221(2):514-20. | used median in the unit |
| Lee SP, Dart AM, Walker KZ, O'Dea K, Chin-Dusting JP, Skilton MR. Effect of altering dietary n-6:n-3 PUFA ratio on cardiovascular risk measures in patients treated with statins: a pilot study. Br J Nutr. 2012 Oct;108(7):1280-5. | used median in the unit |
| Bot M, Carney RM, Freedland KE, Rubin EH, Rich MW, Steinmeyer BC, Mann DL. Inflammation and treatment response to sertraline in patients with coronary heart disease and comorbid major depression. J Psychosom Res. 2011 Jul;71(1):13-7. | used median in the unit |
| Moertl D, Hammer A, Steiner S, Hutuleac R, Vonbank K, Berger R. Dose-dependent effects of omega-3-polyunsaturated fatty acids on systolic left ventricular function, endothelial function, and markers of inflammation in chronic heart failure of nonischemic origin: a double-blind, placebo-controlled, 3-arm study. Am Heart J. 2011 May;161(5):915.e1-9. | used median in the unit |
| Heidarsdottir R, Arnar DO, Skuladottir GV, Torfason B, Edvardsson V, Gottskalksson G, Palsson R, Indridason OS. Does treatment with n-3 polyunsaturated fatty acids prevent atrial fibrillation after open heart surgery?. Europace. 2010 Mar;12(3):356-63. | no inflammatory biomarkers |
| Troseid M, Arnesen H, Hjerkinn EM, Seljeflot I. Serum levels of interleukin-18 are reduced by diet and n-3 fatty acid intervention in elderly high-risk men. Metabolism. 2009 Nov;58(11):1543-9. | used median in the unit |
| Sanders TA, Lewis F, Slaughter S, Griffin BA, Griffin M, Davies I, Millward DJ, Cooper JA, Miller GJ. Effect of varying the ratio of n-6 to n-3 fatty acids by increasing the dietary intake of alpha-linolenic acid, eicosapentaenoic and docosahexaenoic acid, or both on fibrinogen and clotting factors VII and XII in persons aged 45-70 y: the OPTILIP study. Am J Clin Nutr. 2006 Sep;84(3):513-22. | no inflammatory biomarkers |
| Seierstad SL, Seljeflot I, Johansen O, Hansen R, Haugen M, Rosenlund G, FrÃ¸yland L, Arnesen H. Dietary intake of differently fed salmon; the influence on markers of human atherosclerosis. Eur J Clin Invest. 2005 Jan;35(1):52-9. | used median in the unit |
| Geelen A, Brouwer IA, Schouten EG, Kluft C, Katan MB, Zock PL. Intake of n-3 fatty acids from fish does not lower serum concentrations of C-reactive protein in healthy subjects. Eur J Clin Nutr. 2004 Oct;58(10):1440-2. | healthy participants |
| Berstad P, Seljeflot I, VeierÃ¸d MB, Hjerkinn EM, Arnesen H, Pedersen JI. Supplementation with fish oil affects the association between very long-chain n-3 polyunsaturated fatty acids in serum non-esterified fatty acids and soluble vascular cell adhesion molecule-1. Clin Sci (Lond). 2003 Jul;105(1):13-20. | no inflammatory biomarkers |
| Lervang HH, Schmidt EB, MÃ¸ller J, Svaneborg N, Varming K, Madsen PH, Dyerberg J. The effect of low-dose supplementation with n-3 polyunsaturated fatty acids on some risk markers of coronary heart disease. Scand J Clin Lab Invest. 1993 Jul;53(4):417-23. | used median in the unit |
| Zhao YT, Shao L, Teng LL, Hu B, Luo Y, Yu X, Zhang DF, Zhang H. Effects of n-3 polyunsaturated fatty acid therapy on plasma inflammatory markers and N-terminal pro-brain natriuretic peptide in elderly patients with chronic heart failure. J Int Med Res. 2009 Nov-Dec;37(6):1831-41. | omega-3 < 1g per day |
| Erkkila AT, Schwab US, de Mello VD, Lappalainen T, Mussalo H, Lehto S, Kemi V, Lamberg-Allardt C, Uusitupa MI. Effects of fatty and lean fish intake on blood pressure in subjects with coronary heart disease using multiple medications. Eur J Nutr. 2008 Sep;47(6):319-28. | omega-3 < 1g per day |
| Elwakeel NM, Hazaa HH. Effect of omega 3 fatty acids plus low-dose aspirin on both clinical and biochemical profiles of patients with chronic periodontitis and type 2 diabetes: a randomized double blind placebo-controlled study. J Periodontal Res. 2015 Dec;50(6):721-9. | used with aspirin |
| Mani UV, Mani I, Biswas M, Kumar SN. An open-label study on the effect of flax seed powder (Linum usitatissimum) supplementation in the management of diabetes mellitus. J Diet Suppl. 2011 Sep;8(3):257-65. | omega-3 derived from alpha-linolenic acid |
| Foster M, Petocz P, Samman S. Inflammation markers predict zinc transporter gene expression in women with type 2 diabetes mellitus. J Nutr Biochem. 2013 Sep;24(9):1655-61. | omega-3 derived from alpha-linolenic acid |
| Moosheer SM, WaldschÃ¼tz W, Itariu BK, Brath H, Stulnig TM. A protein-enriched low glycemic index diet with omega-3 polyunsaturated fatty acid supplementation exerts beneficial effects on metabolic control in type 2 diabetes. Prim Care Diabetes. 2014 Dec;8(4):308-14. | not RCT |
| Labonte M, Couture P, Tremblay AJ, Hogue JC, Lemelin V, Lamarche B. Eicosapentaenoic and docosahexaenoic acid supplementation and inflammatory gene expression in the duodenum of obese patients with type 2 diabetes. Nutr J. 2013 Jul 15;12:98. | used median in the unit |
| Myrup B, Rossing P, Jensen T, Parving HH, HÃ¸lmer G, Gram J, Kluft C, Jespersen J. Lack of effect of fish oil supplementation on coagulation and transcapillary escape rate of albumin in insulin-dependent diabetic patients with diabetic nephropathy. Scand J Clin Lab Invest. 2001;61(5):349-56. | used OR and 95% CI |
