# Supplementary material for: Omega-3 Fatty Acids Effects on Inflammatory Biomarkers and Lipid Profiles among Diabetic and Cardiovascular Disease Patients: A Systematic Review and Meta-Analysis
Source: Sci Rep. 2019 Dec 11;9:18867. doi: 10.1038/s41598-019-54535-x (PMC6906408; doi:10.1038/s41598-019-54535-x)
Supplement: Supplementary file 7 — S7 Studies chractristics [file 41598_2019_54535_MOESM7_ESM.docx]

**S7: Studies characteristics**

**Omega-3 Fatty Acids Effects on Inflammatory Biomarkers and Lipid Profiles among Diabetic and Cardiovascular Disease Patients: A Systematic Review and Meta-Analysis**

*Zuhair S. Natto BDS, MPH, MSc, DrPH, Wael Yaghmoor*  *BDS, MSc , Heba K. Alshaeri PharmD, MPH, PhD & Thomas E. Van Dyke DDS, MS, PhD.*

Table 1 Omega-3 fatty acids

| **Study** | **Country** | **Test** | **control** | **age** | **F/M** | **duration** | **target** | **outcome** |
| --- | --- | --- | --- | --- | --- | --- | --- | --- |
| Valdivielso et al, 2009 | Spain | 4 capsules of prescription omega 3 fatty acids daily (4g), containing 460 mg of EPA and 380 mg of DHA ethyl esters. | Diet plus Fluvastatin 80 mg Or  Diet 600 kcal below the basic calorie needs | 57 ±5 | 3/5 | 8 weeks | Patients with type 2 diabetes and mixed hyperlipidemia | The intestinal lipoproteins were quantified by the fasting concentration of apolipoprotein B48 using a commercial ELISA |
| Mori et al, 1990 | Australia | 5 capsules 3 times/day with meals. This supplement provided a daily intake of 2.7 g EPA, 1.7 g (DHA), and a total of 4.9 g omega-3 FAs. | No supplements | 33 ± 2 | 0/22 | 6 weeks | insulin-dependent diabetic (IDDM) men | serum cholesterol lipoproteins and apolipoproteins |
| Hilpert etl al, 2007 | USA | 60% high oleic safflower oil, 25% safflower oil, and 15% sardine oil and provided 2.8 g EPA (20:5n–3), 1.2 g DHA (22:6n–3), and 0.2 g ALA | 2,100 mg of sunflower oil | 53.6 ± 1.9 | 5/10 | 4 weeks | Adults with type 2 diabetes | Apo B, LpB, LpB:C, LpB:E LpB: C:E, and LpA-II:B:C:D:E were measured at baseline and 2 and 4 h after the meal. Flow-mediated dilation was measured at baseline and 4 h after the meal. |
| Wong et al, 2010 | China | 4g/day (2% EPA and 25% DHA) | 4g/day of Olive Oil | 61.2 ± 9.0 | 54/43 | 12 weeks | Type 2 DM patients without prior cardiovascular disease | Brachial artery flow-mediated dilation (FMD) and circulating levels of EPCs, metabolic parameters, high-sensitivity C-reactive protein (hsCRP), oxidative stress markers and renal function |
| Lee et al, 2014 | USA | 9 capsules/day = 18% EPA (3.58g) + 12.3% DHA (2.44g) | 9 capsules/day of Corn Oil | Mean 59.9 | 35/24 | 8 weeks | Subjects with early-stage T2D and metabolic syndrome | Levels of serum fatty acids and other serum lipids (triglycerides and total, HDL and LDL cholesterol), markers of inflammation (leptin, and C-reactive protein), as well as glucose regulation (glucose and hemoglobin A1c) |
| Pooya et al, 2010 | Iran | Total daily dose of 2714 mg per day (EPA = 1548 mg; DHA = 828mg and 338 mg of other omega-3 fatty acids) | 2100 mg of sunfloweroil | 56.38 ± 9.24 | Total 81 | 2 months | Type 2 DM patients | Levels of HbA1c, homocysteine, malondialdehyde (MDA) , C-reactiveprotein(CRP), total cholesterol, LDL-cholesterol and fasting blood sugar(FBS) |
| Malekshahi Moghadam et al,2012 | Iran | 2,714 mg of omega-3 fatty acids per day (EPA 1,548 mg; DHA 828 mg; and other omega-3 fatty acids 338 mg) | 2100 mg of sunfloweroil | 55.36 ± 9.88 | 42/42 | 8 weeks | Iranian type 2 diabetes mellitus patients | The serum levels of CRP, IL-2 and TNF-α |
| Hendra et al, 1990 | UK | Twice a day of five MaxEPA capsules (each containing 1 g fish oil) = 1.8 g (EPA) and 1.2 g (DHA) daily | Twice a day of 5 capsules of Olive oil | Mean 56 | 25/55 | 6 weeks | Non-insulin-dependent diabetic (NIDDM) subjects | Hemostatic function and fasting lipid and glucose levels |
| Mansoori et al, 2015 | Iran | 4 soft gels per day (containing 2400 fish oil; DHA: 1450 mg and EPA: 400 mg) | 2400 mg paraffin oil | 55.8 ± 7.6 | Total 68 | 8 weeks | Patients with type 2 diabetes | PPARg-responsive genes related to lipid metabolism |
| Krantz et al,2015 | USA | 465 mg of EPA and 375 mg of docosahexaenoic acid (DHA) for a total daily dose of 3.36 g | Identically matched corn-oil placebo | 61.1 ± 10.3 | 40/22 | 3 months | Hypertension patients | Arterial stiffness measured by pulse wave velocity (PWV) and serum markers of inflammation among patients with hypertension. |
| Root et al, 2013 | USA | 350 mg eicosapentaenoic acid (EPA) and 230 mg docosahexaenoic acid (DHA) per single-dose packet. The oils were emulsified products provided in three premeasured packets | 1.0 g of safflower oil per packet. The oils were emulsified products provided in three premeasured packets; | 21.4 ± 2.9 | 24/33 | 4 weeks | vascular risk factors | Hemodynamic measures (central pulse wave velocity, augmentation index, and aortic systolic blood pressure), inflammatory cytokines (IL-6, IL-8, IL-10, and tumor necrosis factor-α), red blood cell and plasma phospholipid fatty acid profiles, fasting serum lipids, glucose, and C-reactive protein |
| Tinker et al 1999 | USA | 13.2 g fish oil [3.7 g EPA, 1.5 g DHA] | 13.8 g monounsaturated fat source (8.9 g high oleic safflower and soy oil) | NA | total 12 | 6 weeks | CVD risk factors | Postprandial plasma triacylglycerol (TG) and TG-rich lipoprotein (TRL) TG apo B48, and B100 |
| Barbir et al, 1992 | UK | 5g twice daily. Each 1g capsule of Maxepa contains the following active ingredients: Eicosapentaenoic Acid (EPA) 170mg and Docosahexaenoic Acid (DHA) 115mg. |  | 53 + 7 | 9/78 | 3 months | cardiac transplant recipients | the treatment of hyperlipidemia in cardiac transplant recipients. We also compared the effects of both drugs on hemostatic, hematologic and biochemical variables |
| Doenyas-Barak et al, 2012 | Israel | Two pills. Each pill contained 542 mg eicosapentaenoic acid (EPA), 408 mg docosahexanoic acid (DHA), and 2.75 IU vitamin E | 1ml of soya oil for 3 weeks | 59±7 | 15/17 | 20 weeks | hypercholesterolemic patients | Platelets function of hypercholesterolemic patients. Blood pressure (BP) and heart rate (HR), endothelial function, lipid profile, interleukin (IL)-6 and oxidative stress (STAT-8-Isoprostane) were concomitantly assessed |
| de Mello et al,2009 | Finland | Baltic herring, whitefish, vendace, or tuna (EPA+DHA from fish: =2 g/day) | lean meat (beef or pork) or skinless chicken, less than 1 fish meal per week) | 62.7 ± 6.3 years | Total 27 | 8 weeks | subjects with CHD | inflammatory and endothelial function-related genes in peripheral blood mononuclear cells (PBMCs) of subjects with CHD, and its association with serum fatty acid (FA) profile and lipid metabolic compounds |
| Mehra et al, 2006 | USA | 8 g/day. 1 g of the n-3 ethylester concentrate was shown to consist of 80% n-3 fatty acid ethyl esters (44% eicosapentaenoic acid [EPA], 24% docohexaenoic acid [DHA], 12% other n-3 fatty acid ethyl esters). | 8g of Corn oil / day | 57 + 12 years | 4/10 | 18 weeks | patients with advanced heart failure | pro-inflammatory cytokines (TNF-a, IL-1) and body weight in patients with advanced heart failure |
